# Impedance spectroscopy as a tool to monitor the adsorption and removal of nitrate ions from aqueous solution using zinc aluminum chloride anionic clay

**DOI:** 10.1016/j.heliyon.2018.e00536

**Published:** 2018-03-01

**Authors:** A. Elmelouky, A. Mortadi, El. Chahid, R. Elmoznine

**Affiliations:** Laboratory Physics of Condensed Matter (LPMC), University Chouaib Doukkali, El-Jadida, Morocco

**Keywords:** Materials science, Materials chemistry

## Abstract

In this study, Zn_3_AlCl ionic clay was used to investigate the adsorption mechanism of the nitrate ions in solutions containing nitrate ions at different contact time. The clay was synthesized by coprecipitation method at room temperature, and this sample was characterized by XRD, Fourier transform-infrared (FT-IR) and inductively coupled plasma (ICP). This sample was crystallized in a rhombohedral symmetry (Space group: R-3 m). Impedance spectroscopy was used as a tool to evaluate and monitor the adsorption process at different contact time 5; 10; 20; 30; 60 min and the clay alone. The impedance measurement was well analyzed and fitted with an equivalent circuit containing both (R//CPE) connected in series. Furthermore, the σ_ac_ conductivity was also investigated as a function of frequency. It was analyzed and fitted using double power law: $\sigma_{ac}\left(\omega \right)=\sigma_{dc}+A\omega^{s1}+B\omega^{s2}\text{,}\left(0\leq {\text{s}}_{1}\leq 1\;\text{and}\;0\leq {\text{s}}_{2}\leq 1\right)\text{.}$

This study reveals the existence of two relaxation processes with different relaxation times, which could be attributed to the grain and grain boundaries, and exhibit high values of dielectric constant at low frequencies.

## 1 Introduction

The contamination of wastewater by nitrate ions has become an ever increasing and serious environmental threat [1]. The excessive application of the chemical product in industrial sectors causes the increases of large quantities of these ions into wastewater and surface water [2]. The solubility of nitrate ions is very high in wastewater [3]. It could be considered as the most widespread contaminant, and it exhibits a serious threat to drinking water supplies and promoting eutrophication [4, 5]. The higher amount of nitrate in drinking water can also cause several problems such as gastric cancer [6].

The methemoglobinemia or blue baby syndrome, a serious health risk, occurs when nitrate is converted to nitrite, which then reacts with the hemoglobin to cause blueness of the skin of newborn infants [7]. The high costs of adsorption using adsorbents such as activated carbon lead to the researchers to find other cheaper substituents such as lamellar double hydroxides (LDH). This later has been shown to be effective for the removal of this contaminant from industrial washing water [8]. As it is known, lamellar double hydroxides (LDH) can be described by the general formula [M^2+^_1-x_, M_x_^3+^, (OH)_2_]^x^^+^ [X^m^^−^_x/m_, nH_2_O] where M^2+^ and M^3+^ represent divalent and trivalent metal cations, respectively, x+ represents a trivalent cation metal; X^m−^ represents the anion intercalated in the interlamellar space [9]. The advantage of using LDH is the easy modification of their properties by varying the composition of the sheet and the intercalated anions [10], which subsequently allows them to increase their capacity to contaminants retention from the various industrial discharges. Therefore, the present study aims to synthesize and study the nitrate removal efficiency by Zn_3_AlCl. The complex impedance spectroscopy was employed to investigate the microstructural changes of the electrical properties of Zn_3_AlCl that could occur during the adsorption process.

## 2 Experimental

### 2.1 Materials

At constant pH the anionic clay (Zn_3_AlCl) were synthesized by coprecipitation method [11]. This sample were prepared at ratio: [Zn^2+^]/[Al^3+^] = 3. The Zn_3_AlCl was been in contact for 5, 10, 20, 30 and 60 min with a potassium nitrate solution (400 mg/L). After each adsorption time, the adsorbate is filtered and the precipitate (LDH and retained nitrate) is recovered and dried in an oven at 50 °C for 48 hours.

### 2.2 X-RAY diffraction

Powder X-ray diffraction (PXRD) patterns of the samples were recorded on a X-ray diffractometer (SIEMENS D 501) (λK_α1_ = 1.5405 Å and λK_α2_ = 1.5444 Å) radiation. The XRD pattern was carried out under the following operating conditions:

- Domain angles in 2θ: 2°–76°.
- Increasing angle in 2θ: 0.08°.
- Integration time by counting: 4s.

### 2.3 Fourier transform-infrared

Infrared measurements were performed with a Perkin-Elmer 16 PC Fourier Transform Spectrometer (FTS). The samples were prepared in the pellet of a 13 mm diameter and 1mm thickness using 2 mg of product diluted in 200 mg of KBr. The FT-IR spectra were recorded in absorbance in the wave number range of 400–4000 cm^−1^ at room temperature with a resolution of 1 cm^−1^.

### 2.4 Induced coupled plasma (ICP) measurements

The metal ratio was determined using ICP measurements. A gas or plasma consisting of ions, electrons and neutral particles is formed from the argon gas, which is then used to atomize and ionize the components of the sample. The results of elemental analyses by ICP led to an average composition for Zn_3_AlCl sample which corresponds to the chemical formula:

(Zn_2.93_Al(OH)_7.86_) (Cl^−^, 1.87H_2_O), this formula is found by [12,13].

### 2.5 Impedance spectrscopy measurements

The impedance spectroscopy measurements were performed in the frequency range from 20 Hz to 1 MHz with eight points per decade at room temperature, using an impedance analyzer (Hewlett Packard, 4192 A).

The electrical contacts were performed by using silver electrodes, which were deposited on the two circular faces of the sample. The magnitude of the applied signal is 0.6 V peak to peak. An amount of 200 mg is pelleted to make the analyzes impedance. The granulated powder was compacted under a hydraulic press with 250 MPa pressure into discs of 13 mm diameter and of 1 mm thickness approximately. The impedance spectra were recorded at different adsorption time (5; 10; 20; 30; 60 min).

Analysis and fitting of impedance spectra with complex empirical functions were carried out using commercial Zview® software.

## 3 Results and discussion

### 3.1 Structural study

**Fig. 1:**
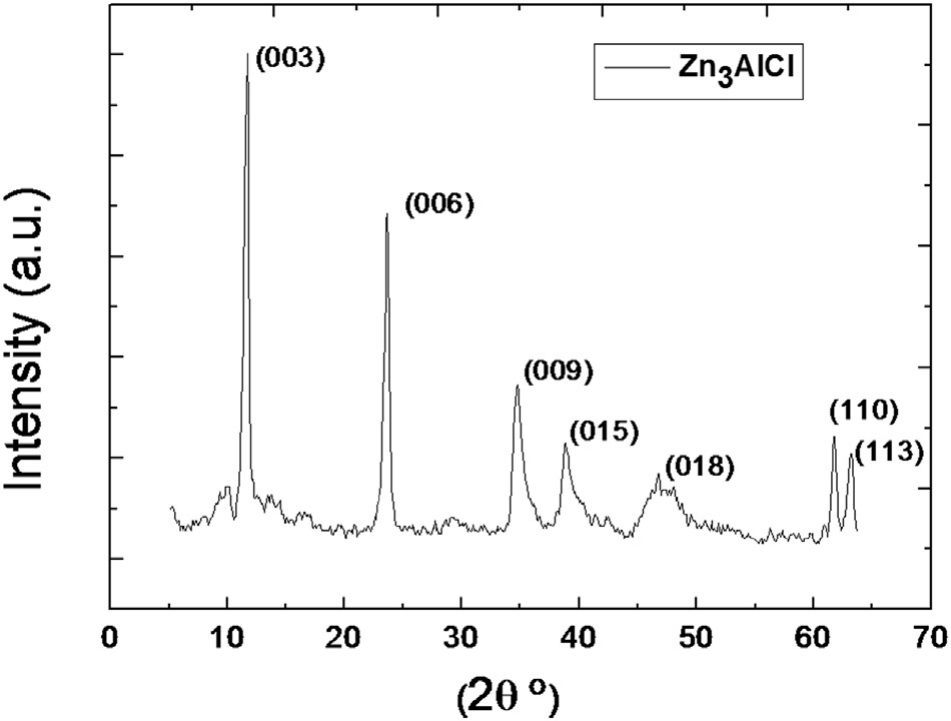
XRD pattern of Zn_3_–Al–Cl

The X-ray diffraction patterns of Zn_3_AlCl shown in (Fig. 1) of the sample is characteristic to those a double lamellar hydroxide. The sample was crystallized in a rhombohedral symmetry (space group: R-3 m) with: (c/3) = d_003_ = 2.d110 and a (intermetallic distance) = 2d_006_. The lattice parameters **c** and **a** are respectively 2.38 and 0.31 nm. These values are in agreement with those reported in the literature [14].

### 3.2 Infrared diagram

**Fig. 2:**
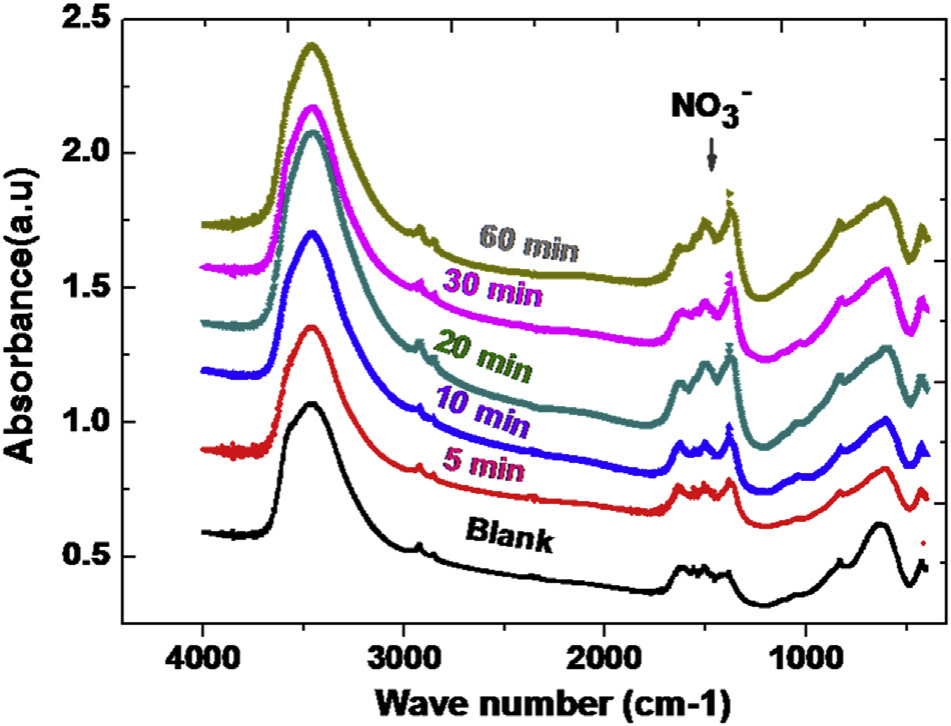
FT-IR spectrum of peak corresponds of nitrate ions adsorption

Fig. 2 shows the IR spectra of Zn_3_AlCl-NO_3_^−^ at different contact time. The variation in these spectra indicates the adsorption of the ions nitrate by anionic clay. The IR spectrum shows a significant change in the absorbance magnitude when nitrate ions are adsorbed on the clay surface. The clay has a very significant adsorption efficiency. This shows a very effective surface effect for trapping the nitrate ions during the contact time.

Infrared spectra in the 400–4000 cm^−1^ range show a typical pattern of the LDH phase. Indeed, the spectra of the LDH generally present three characteristic domains [15], between 3400 and 3600 cm^−1^, appear the vibration bands of the hydroxyl groups of the sheets ν(OH) and physisorbed or intercalated water molecules [16]. At 1600 cm^−1^ of the intercalated water molecules and thus adsorbed the intense band to the corresponding 1380 cm^−1^ of the nitrate ion. For the low frequencies (400–1000 cm^−1^), the bands due to network vibrations appear υ (M-O) and δ (O-M-O). In Table 1 we have reported the essential vibration waves.

**Table 1:** The Characteristic bands of XRD for the Zn_3_AlCl

Sample LDH	Ratio	*v* (OH) (cm^−1^)	δ (Η_2_Ο) (cm^−1^)	ν (M–O) (cm^−1^)	δ (O–M–O) (cm^−1^)
Zn_3_AlCl	R = 3	3454.6	1628	613.38	426.28

### 3.3 Impedance spectroscopy analysis

#### 3.3.1 Cole-Cole plot

**Fig. 3:**
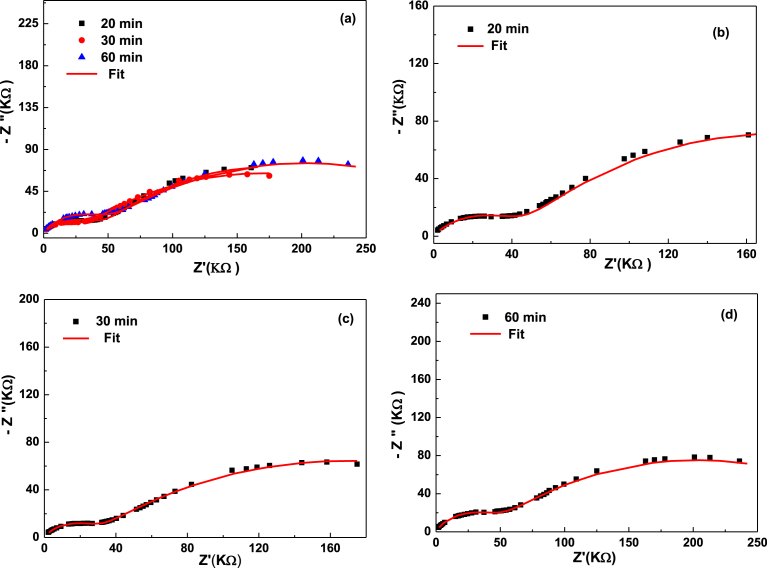
**Z**″ as a function of Z′ at different contact time: 20; 30; 60 min (a). (b), (c) and (d) represent the Nyquist diagram for 20; 30 and 60 respectively. Solid line correspond to the fit.

**Fig. 4:**
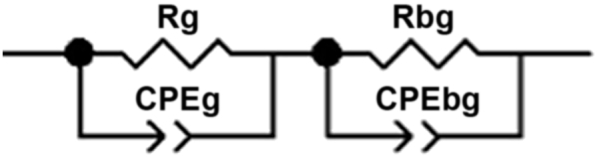
Equivalent circuit that modelize the impedance spectra of Zn_3_AlCl during NO_3_^−^ ions adsorption.

The impedance spectra obtained at different times of adsorption are presented in Figs. 3 and 4. They show the plot of (−Z″) versus (Z′) taken over the frequency range of 20 Hz–1 MHz at different times (5 min, 10 min, 20 min, 30 min, 60 min). These plots exhibit depressed semicircles having centers lying below the real axis confirming the presence of the non-Debye type of relaxation phenomenon in the materials [17]. All samples show two semicircles are attributed to two relaxation process, one located at higher frequency represent the grain effect and other is located at the medium frequency is attributed to the grain boundary. The impedance spectra are modeled using the equivalent circuit, the circuit is composed of two blocs serially connected (R//CPE) which describe respectively grains (intra-granular) and grain-boundaries (inter-granular) effects. However, reports have been found in the literature on electric modulus [18]. In contrast, the impedance spectroscopy of LDH is scarce studied. For this reason, an in-depth study was carried out in this work.

Several authors [19], are attributed each semicircle an RC parallel circuit. In reality, the capacitance was replaced by a constant phase element (CPE) used for describing a distributed charge accumulation on the rough, irregular LDH surface [12, 20], when the nitrate ions began to adsorb at the LDH surface.

**Fig. 5:**
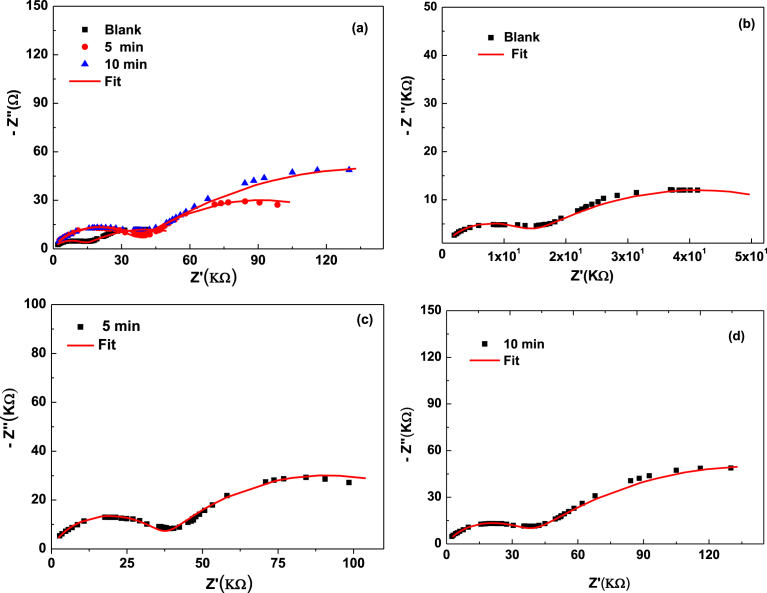
Z″ as a function of Z′ at different contact time: 0; 5; 10 min (a). (b), (c) and (d) represent the Nyquist diagram for 0; 5 and 10 min respectively. Solid line correspond to the fit.

In our case, the appearance of two semicircles (Figs. 3 and 5), suggests the presence of both bulks (grain) as well as grain boundaries effects. Fig. 5 shows the equivalent circuit model used for the according of all samples.

The expression of the complex impedance of the sample is:

(1) $$Z^{*}\left(\omega \right)=Z_{g}^{*}\left(\omega \right)+Z_{bg}^{*}\left(\omega \right)$$

The impedances of grains and grain boundaries represented by the following expression:

(2) $$Z^{*}\left(\omega \right)=\frac{R_{g}}{1+{\left(j\tau_{g}\cdot \omega \right)}^{p_{g}}}+\frac{R_{bg}}{1+{\left(j\tau_{bg}\cdot \omega \right)}^{p_{bg}}}$$

The complex impedance of a system at an applied frequency can be written as sum of the real and imaginary parts:

(3) $${\text{Z}}^{*}\left(\omega \right)=\text{Z}’\left(\omega \right)+\text{jZ}’’\left(\omega \right)$$

Where Z′ and Z″ are the real part and the imaginary part respectively of the complex impedance and ω is the angular frequency.

The real part of the complex impedance indicates the contribution of grains and boundaries is given by

(4) $$Z^{\prime }\left(\omega \right)=\frac{Rg{\left(\tau_{g}\omega \right)}^{pg}\;\cos\left(\frac{p_{g}\pi }{2}\right)+R_{g}}{{\left[1+{\left(\tau_{g}\omega \right)}^{pg}\;\cos{\left(\frac{p_{g}\pi }{2}\right)}^{2}+{\left(\tau_{g}\omega \right)}^{pg}\;\sin\left(\frac{p_{g}\pi }{2}\right)\right]}^{2}}+\frac{R_{jg}{\left(\tau_{jg}\omega \right)}^{pjg}\;\cos\left(\frac{\pi \cdot pjg}{2}\right)+Rjg}{{\left[1+{{\left(\tau_{jg}\omega \right)}^{p_{jg}}\;\cos\left(\frac{pjg\cdot \pi }{2}\right))}^{2}+({\left(\tau_{jg}\omega \right)}^{pjg}\sin\left(\frac{p_{jg}\pi }{2}\right)\right]}^{2}}$$

The imaginary part of the complex impedance indicates the contribution of grain and grain boundaries is given by:

(5) $$Z^{''}\left(\omega \right)=\frac{R_{g}{\left(\tau_{g}\omega \right)}^{p_{g}}\;\sin\;\left(\frac{p_{g}\pi }{2}\right)}{{\left[1+{\left(\tau_{g}\omega \right)}^{p_{g}}\;\cos{\left(\frac{p_{g}\pi }{2}\right))}^{2}+({\left(\tau_{g}\omega \right)}^{p_{g}}\;\sin\left(\frac{p_{g}\pi }{2}\right)\right]}^{2}}+\frac{R_{jg}{\left(\tau_{jg}\omega \right)}^{p_{jg}}\;\sin\;\left(\frac{p_{jg}\pi }{2}\right)}{{{\left[1+{\left(\tau_{jg}\omega \right)}^{p_{jg}}\;\cos{\left(\frac{\pi p_{jg}}{2}\right))}^{2}+({\left(\tau_{jg}\omega \right)}^{p_{jg}}\;\sin\left(\frac{p_{jg}\pi }{2}\right)\right]}^{2}}^{2}}$$

Two clear time of relaxation in impedance Cole-Cole plot have also been successfully explained by employing two parallel (R//CPE).

Nyquist plot show each block is given a single relaxation time of the grains and grains boundaries, respectively.

Where ${\text{τ}}_{\text{i}}={\left(R_{i}T_{i}\right)}^{1/p_{i}}$, i = grain (g), grain boundaries (bg).

The first term of the (Eq. (5)) of the imaginary part (Z″) modeled the grain effect has a maximum which occurs at:

(6) $${\left(\omega_{\max}\right)}_{g}=\tau_{g}^{-1}={\left(R_{g}T_{g}\right)}^{\frac{1}{p_{g}}}$$

The second term of the (Eq. (5)) of the imaginary part (Z**″**) modeled the grain boundary effect has a maximum that occurs:

(7) $${\left(\omega_{\max}\right)}_{bg}=\tau_{bg}^{-1}={\left(R_{bg}T_{bg}\right)}^{\frac{-1}{p_{bg}}}$$

Therefore, the high frequency semicircle indicates the grain effect [21] and the grain boundaries effect occurs at medium frequency [22, 23]. From each semicircle observed, the characteristic parameters derived for a given time are:

- The resistance R_i_, which is the point of intersection of the semicircle considered with the real axis.
- P_i_ dispersion coefficient and T_i_ pseudo capacitance and the constant phase element (CPE).
- Time (τ_i_) of relaxation is obtained using equation: $\left({\text{τ}}_{\text{i}}={\left({\text{R}}_{\text{i}}{\text{T}}_{\text{i}}\right)}^{1/{\text{p}}_{\text{i}}}\right)$ [12]. The resistance of grains boundaries is greatly influenced by the adsorption phenomena, with increasing NO_3_^−^ content adsorbed, the diameter of these semi-circular arcs changes systematically which is an indication of the relative contributions from grains boundaries resistance and grains resistance. The values of the fitted equivalent circuit parameters have been evaluated and listed in Table 2.

**Table 2:** The values of the fitted equivalent circuit parameters of grains (g), boundaries grains (bg).

Time (min)	σg (μS/m)	τg (ms)	Cg (nF)	σjg (μS/m)	τjg (μS)	Cjg (nF)
0	8.68	3.89	0.63	1.62	1.10	1.01
5	2.77	7.86	0.71	0.78	1.69	0.60
10	2.95	21.16	0.78	0.37	2.02	0.75
20	3.04	28.26	0.70	0.25	2.58	0.98
30	3.76	7.20	0.55	0.34	2.85	0.79
60	2.29	3.86	0.12	0.31	3.35	0.89

**Fig. 6:**
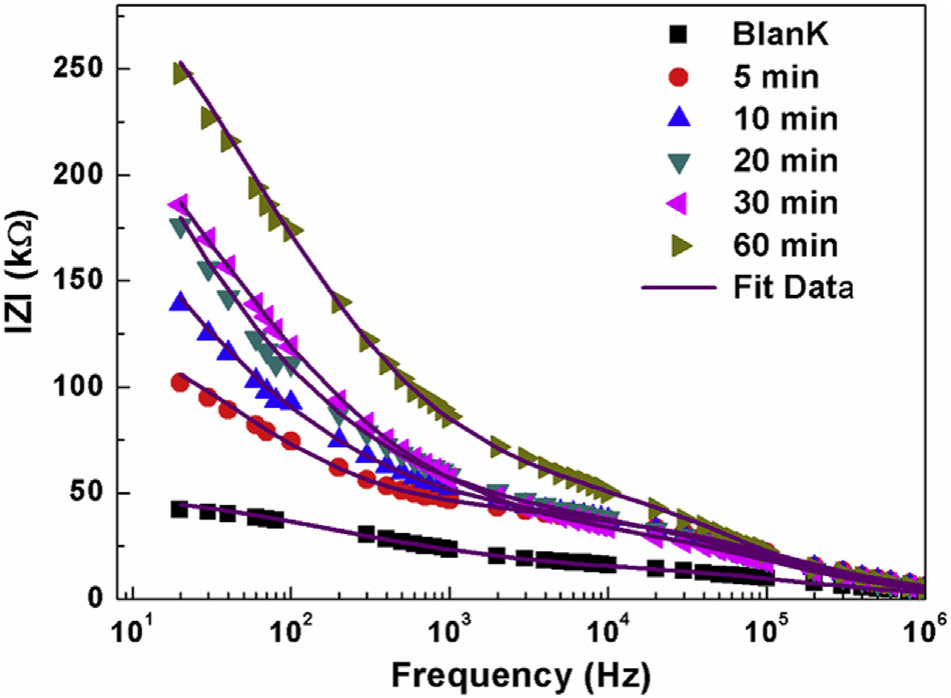
Frequency dependence of |Z| for each time of adsorption with fit by circuit

Fig. 6 shows the variation of the impedance magnitude as a function of frequency at different contact time. The impedance magnitude decreases as a function of frequency. All samples show a similar behavior, at medium frequency the impedance modulus decreases rapidly with increasing frequency, thus the impedance magnitude increase with the contact time increase; at higher frequency the evolutions of the impedance modulus are independent of the frequency and the contact time. The change observed in these variations indicates that the amount of the absorbed nitrate ions increases with the contact time.

#### 3.3.2 Dielectric properties

The LDH are heterogeneous solids that have low mobility charge carriers. LDH have two carriers which are responsible for the dielectric response of LDH [20, 24]. The first carrier is the proton of the polarized water cluster in the LDH interlayer (proton transfers create OH^−^and H_3_O^+^ groups at each end of the path) due to the applied electric field. The second carrier is the range of anions (NO_3_^−^) adsorbed in the area of LDH. NO_3_^−^ ions move from their equilibrium positions under the application of an electric field.

The study of the dielectric characterization is an essential source of valuable information about conduction processes, since we can determine the origin of the dielectric loss, the electrical and dipolar relaxation [25]. The dielectric relaxation is described by a non-Debye model which gives the frequency-dependent of complex permittivity in the form [26].

(8) $$\varepsilon^{*}\left(\omega \right)=\varepsilon^{'}\left(\omega \right)-j\varepsilon^{''}\left(\omega \right)=\varepsilon_{{}_{\infty }}+\frac{\varepsilon_{s}-\varepsilon_{\infty }}{1+{\left(j\omega \tau \right)}^{\alpha }}-j\frac{\sigma^{*}}{\omega^{n}\varepsilon_{0}}$$

Where: ɛ_s_, ${\text{ε}}_{\infty }$ are the static permittivity and infinite permittivity of the dielectric constant, respectively, and σ* = σ_1_ + jσ_2_ is the complex conductivity, τ is the relaxation time, α is a constant between 0 and 1.

The real part of complex permittivity ɛ*is given by the flowing relation:

(9) $$\varepsilon^{\prime }\left(\omega \right)=\varepsilon_{\infty }+\frac{\left(\varepsilon_{s}-\varepsilon_{\infty }\right)\left[1+{\left(\omega \tau \right)}^{1-\alpha }\;\sin\left(\alpha \frac{\pi }{2}\right)\right]}{1+2{\left(\omega \tau \right)}^{1-\alpha }\;\sin\left(\alpha \frac{\pi }{2}\right)+{\left(\omega \tau \right)}^{2-2\alpha }}+\frac{\sigma_{2}}{\varepsilon_{0}\omega^{n}}$$

The imaginary part of complex permittivity ɛ* is:

(10) $$\varepsilon^{\prime \prime }\left(\omega \right)=\frac{\left(\varepsilon_{s}-\varepsilon_{\infty }\right){\left(\omega \tau \right)}^{1-\alpha }\;\cos\left(\alpha \frac{\pi }{2}\right)}{1+2{\left(\omega \tau \right)}^{1-\alpha }\;\sin\left(\alpha \frac{\pi }{2}\right)+{\left(\omega \tau \right)}^{2-2\alpha }}+\frac{\sigma_{1}}{\varepsilon_{0}\omega^{n}}$$

Where ɛ_s_ is the static permittivity at low frequency, ε∞ the infinite permittivity at high frequency; ɛ_0_ is the vacuum permittivity, **σ**_**1**_ and **σ**_**2**_ represent the conductivity at low frequency, and the parameter **n** represents the exponent and ω is the angular frequency.

The dielectric constant and the dielectric loss follow the power frequency law as indicated by the equations below [27, 28, 29]:

(11) $$\varepsilon^{'}\approx \varepsilon^{''}\approx \omega^{-p},\;\;\omega <\;\omega_{h\;\;\;\;\;\;\;\;}0<p<1$$

Where ɛ′(ω) and ɛ″(ω) are the real and imaginary parts of the dielectric permittivity, respectively.

ω_h_: is the characteristic frequency. While p < 1 indicates a distribution of carrier polarization mechanism.

The slope of the ɛ″ (ω) at medium frequency is less than 1 which implies the conduction process is not the case for dc-conductivity [30]. In our case 0.8 ≤ p < 1, then the MFD response in LDH material can be ascribed to the hopping of charge carriers between localized sites.

**Fig. 7:**
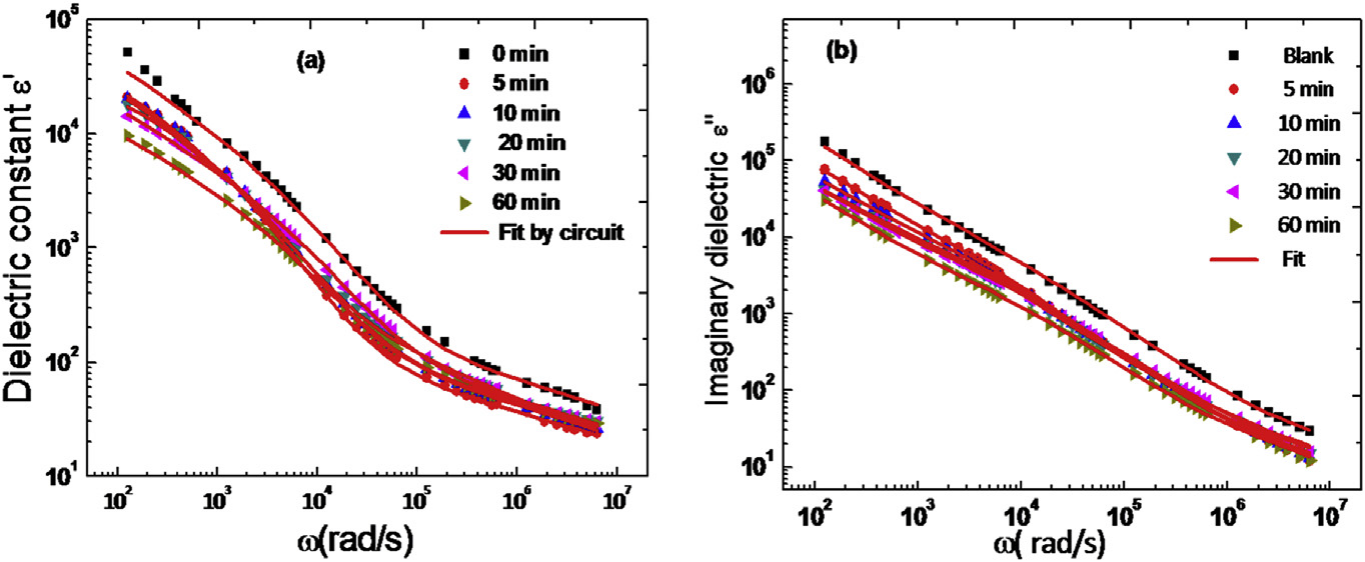
Frequency dependence of dielectric constant ɛ**′** (a) and imaginary dielectric ɛ**″** (b) for each time of adsorption with the fit using the equivalent circuit.

Fig. 7 shows the variation of the real part ɛ′ of the dielectric permittivity, with frequency at different contact time of adsorption and at room temperature. It is observed from this figure that the variation of ɛ′ and ɛ″ decreased almost linearly as a function of the logarithmic frequency, which can be attributed to the anomalous medium frequency dispersion (AMFD) when the current carriers are dominant in the dielectric [20]. The values of ɛ′ and ɛ″ were only slightly affected by the contact time of adsorption of the ions NO_3_^−^ by anionic clay Zn_3_AlCl. In our case the dielectric behavior of LDH at medium frequency can be described also by (AMFD). It was modeled by the second type of universal power law (Eq. (10)) [31, 32, 33]. The dielectric properties of anionic clay (LDH) in both the Nyquist and Bode diagrams will be determined by the relaxation mechanism which can be achieved by either intra-cluster or inter-cluster charge recombination of water molecule, or other routes (NO_3_^−^) as result of applied electric field in the frequency range [34]. The water cluster array is neutral, in the absence of an applied electric field and the LDH sample appears unpolarized.

#### 3.3.3 AC-conductivity

Layered double hydroxides are heterogeneous solids known by their low charge carrier mobility. The conductivity in AC current following the (Eq. (12)) this law allows the adjustment of the experimental data using equivalent circuit, we then find:

(12) $$\sigma_{\mathrm{ac}}\left(\omega \right)=\sigma_{\mathrm{dc}}+A\omega^{s1}+B\omega^{s2}\;\mathrm{with}\;0<s_{1},s_{2}<1$$

**Fig. 8:**
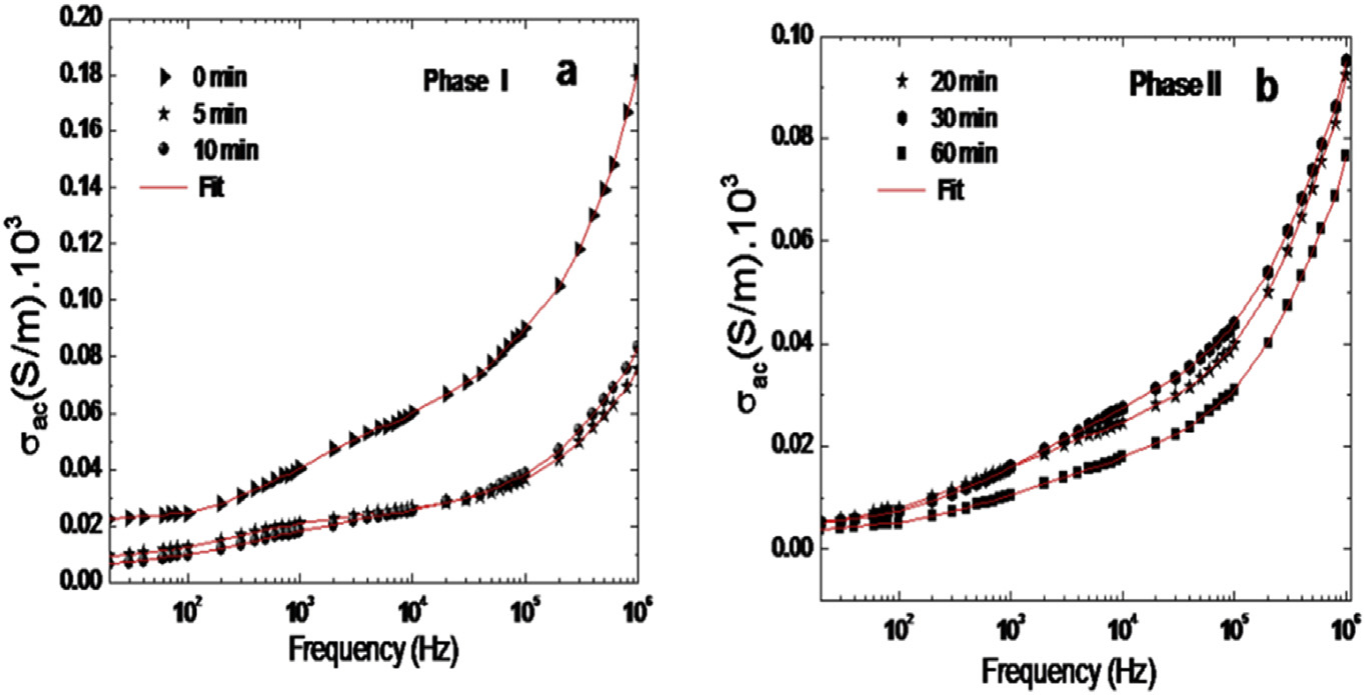
(a) Variation σ_ac_ as a function of phase I and (b) phase II with Frequency at room Temperature for Zn_3_AlCl. Solid line correspond the fit using equivalent circuit.

Fig. 8 shows the frequency dependence of AC conductivity (σ_ac_) at different contact time. The variation of (σ_ac_) increases with increasing frequency, indicating that the electrical conduction in the material is proportional to Aω^s1^ + Bω^s2^. An agreement between the experimental and theoretical results suggests that the AC conductivity behavior of Zn_3_AlCl can be explained by double hopping conduction.

As illustrated in the inset of Fig. 8 a typical conductivity-frequency spectrum is divided into three parts. In region 1, according to the hopping relaxation model, since the frequency is low and the electric field cannot perturb the hopping conduction mechanism of charged particles, the conductance is approximately equal to the dc value and the conduction mechanism is the same as that for dc conduction, i.e., hopping of charged particles from one localized site to another.

**Fig. 9:**
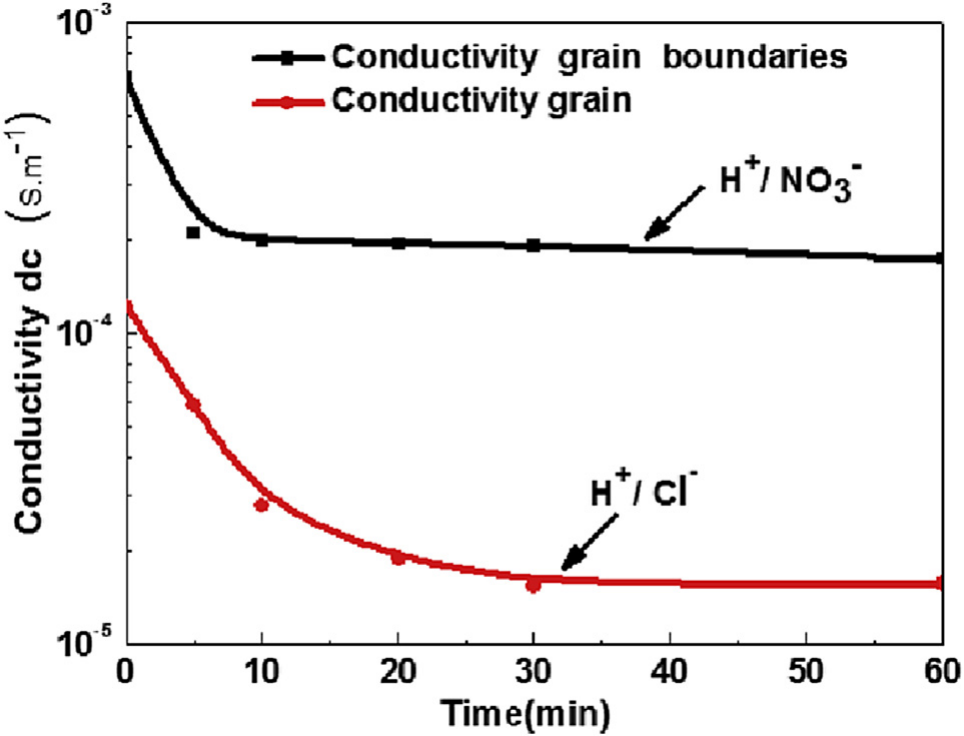
Variation σ_dc_ conductivity of grain boundaries and grain as a function of time contact

Fig. 9 shows the variation of dc conductivity of both grain and grain boundaries. The contribution dc conductivity is dominant in the medium frequency associated to grain boundaries, and in the high frequency associated with grain.

**Fig. 10:**
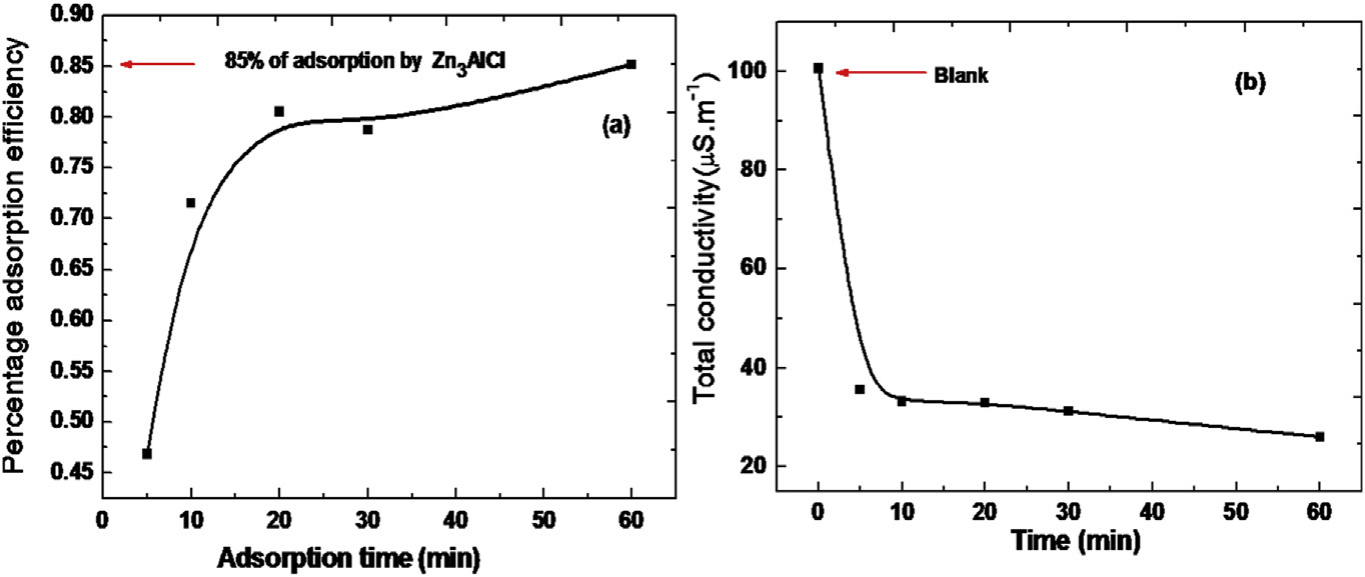
(a) Evolution the efficiency and (b) Evolution the total electrical conductivity of the sample during the adsorption periods. Solid line correspond to the fit using the equivalent circuit.

Fig. 10 (a) and (b) shows the evolution of percentage of adsorption efficiency and the total conductivity respectively as a function of adsorption time. The evolution of percentage of adsorption efficiency shows two phases below and above 20 min. In the first phase the adsorption efficiency increases rapidly with the adsorption time increase. Moreover, the second phase increases slowly. This implies that the majority of ions are adsorbed up to 20 min. This behaviour is confirmed by the measured total conductivity, Fig. 10 (b). The similar behaviour is obtained by other authors in their kinetics studies [35].

## 4 Conclusions

The Impedance Spectroscopy is a very important method for monitoring of adsorption phenomenon, using the Cole-Cole diagram. The adsorption was affected by two regions, e.g. grain and grain-boundaries. Impedance data were analyzed using an equivalent circuit, enabling the determination of grain and grain boundary contributions. At the high quantity of nitrate ions, the grain boundary contribution was dominant during all times of adsorption. The values of R_g_ and R_bg_ were obtained by this formalism. We have found that the retention coefficient arrives at 85% of ions adsorbed by anionic clay.

## Declarations

### Author contribution statement

Abderrahmane Elmelouky, Abdelhadi Mortadi: Conceived and designed the experiments; Performed the experiments; Wrote the paper.

Reddad Elmoznine: Analyzed and interpreted the data; Contributed reagents, materials, analysis tools or data

Elghaouti Chahid: Analyzed and interpreted the data

### Funding statement

This research did not receive any specific grant from funding agencies in the public, commercial, or not-for-profit sectors.

### Competing interest statement

The authors declare no conflict of interest.

### Additional information

No additional information is available for this paper.
